# Ectopic Germinoma: A Delayed Diagnosis With Serial Magnetic Resonance Imaging Features

**DOI:** 10.7759/cureus.20760

**Published:** 2021-12-27

**Authors:** Mohd Imree Azmi, Nurul Hafidzah Rahim, Yee Ping Ang, Sellymiah Adzman, Sarawana Chelwan Muniandy

**Affiliations:** 1 Radiology, Universiti Kebangsaan Malaysia Medical Centre, Kuala Lumpur, MYS; 2 Radiology, Hospital Kuala Lumpur, Kuala Lumpur, MYS; 3 Pathology, Hospital Kuala Lumpur, Kuala Lumpur, MYS

**Keywords:** serial, mri, imaging, ectopic, germinoma

## Abstract

Germinoma arising from intracranial off-midline structures is considered ectopic. Although basal ganglia germinoma is a rare occurrence, it is more commonly seen in the Asian population, particularly among boys. Here, we report a case of an adolescent boy who presented with hemiplegia and delayed diagnosis of basal ganglia germinoma with progression on serial magnetic resonance imaging (MRI). Several signal changes have been described during the early stage of the disease such as T2-weighted patchy hyperintense signal, cerebral hemiatrophy, and signal change on susceptibility-weighted imaging. ^11^C-methionine positron emission tomography is an additional imaging technique that can reveal ectopic germinoma. Follow-up MRI revealed small cystic changes, and the latest imaging showed progression into a large multicystic lesion with mass effect. The patient underwent surgery, and histopathological examination revealed basal ganglia germinoma. We highlight the serial MRI changes that were suggestive of basal ganglia germinoma in this case.

## Introduction

Intracranial germ cell tumors (GCT) are rare tumors that typically occur in midline structures, namely, the pineal gland and the neurohypophyseal region of the brain. The tumor is considered ectopic when it arises from off-midline structures such as the basal ganglia, thalamus, septum pellucidum, and cerebellum. Germinoma is a common subset of GCT with an incidence rate of 0.2 per 1,000,000 individuals [1]. Germinoma arising from the basal ganglia is very rare and is considered ectopic with an estimated occurrence ranging between 4% to 20% of all intracranial germinoma. It is more commonly found in the adolescent Asian population with a high male predominance (male-to-female ratio of 3-15:1 with intracranial germinoma) [2-4]. Diagnosis of early basal ganglia germinoma is challenging as the imaging findings are non-specific and patients may have atypical or vague symptoms at presentation. The typical signs and symptoms include hemiparesis, cognitive decline, and psychosis. Germinoma is a highly radiosensitive tumor with favorable outcomes [5]. Here, we report the case of a patient with a delayed diagnosis of ectopic germinoma with serial imaging findings from the onset of symptoms to a large solid-cystic basal ganglia mass.

## Case presentation

**Figure 1:**
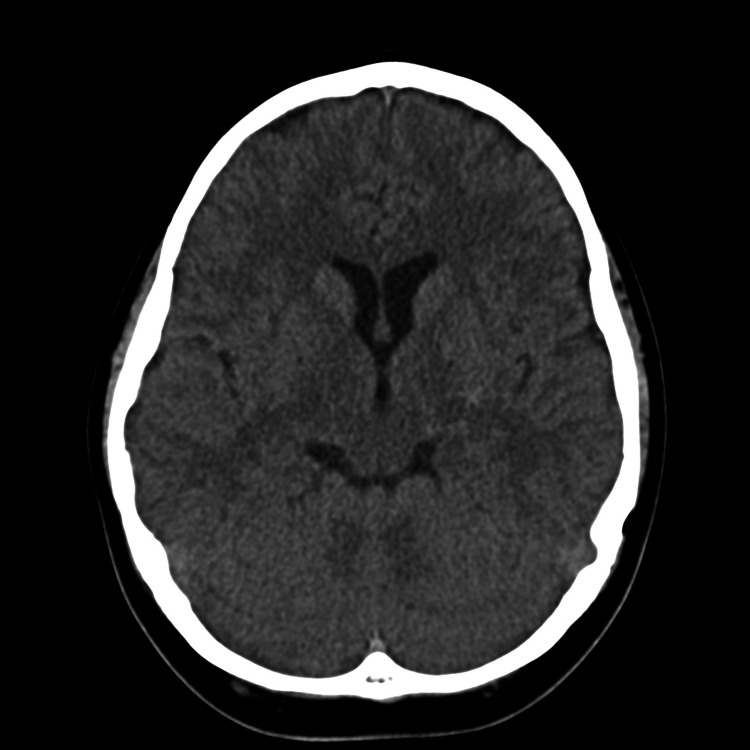
NECT of the brain showing mild density in the region of the left basal ganglia at the posterior limb of the internal capsule. A slight asymmetry of the lateral ventricles with the larger left lateral ventricle indicates possible cerebral hemiatrophy. No mass effect or hydrocephalus can be seen. NECT: non-enhanced computed tomography

**Figure 2:**
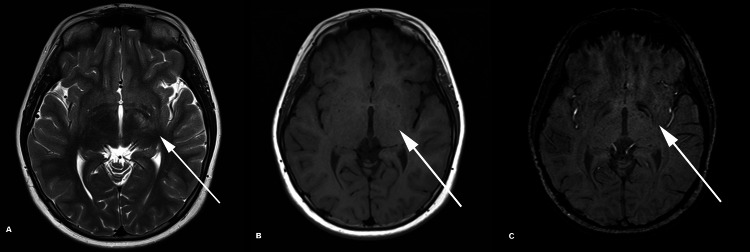
MRI in axial planes showing T2-weighted (A), T1-weighted (B), and SWI images (C). An ill-defined patchy hyperintense signal on T2-weighted (A, white arrow) and an isointense signal for cortical gray matter on T1-weighted images can be seen (B, white arrow). On SWI (C), an associated blooming artifact of the same region can be seen (white arrow). MRI: magnetic resonance imaging; SWI: susceptibility-weighted imaging

**Figure 3:**
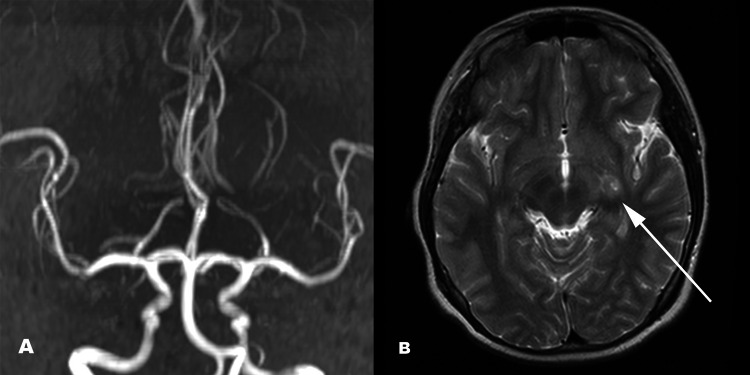
Follow-up MRI showing MR angiography (A) and axial-cut T2-weighted image (B). No stenosis, beaded appearance, or abnormal signal intensity from the vessel can be seen on MRA (A), particularly on the left side. Retrospectively, small cystic lesions were noted in the left basal ganglia (B, white arrow). There was also persistent mild asymmetry of the lateral ventricle, along with a slightly larger left lateral ventricle (not shown). MRA: magnetic resonance angiography

A 14-year old boy with no known medical illness presented with right hemiplegia and an ipsilateral upper motor neuron lesion of the seventh cranial nerve. His symptoms had started three years ago, and, initially, he reported experiencing right-sided body weakness associated with numbness. He also complained of left-sided headache, which he described as a sharp pain. Non-enhanced computed tomography (CT) was normal (Figure 1). On his first magnetic resonance imaging (MRI) in 2017, abnormal signal changes were noted in the left cerebral peduncle and left basal ganglia. Retrospective scrutiny of the initial MRI showed an abnormal hyperintense signal on the T2-weighted image at the left basal ganglia with an isointense signal on the T1-weighted image. Moreover, an associated abnormal signal was noted on susceptibility-weighted imaging (SWI) along with asymmetry of the lateral ventricles, indicative of possible cerebral hemiatrophy (Figures 1, 2). There was no change in his symptoms and a repeat MRI one year later in 2018 showed no significant interval changes. However, closer inspection revealed the development of tiny cystic lesions in the left basal ganglia with a mild increase in the size of the ill-defined patchy T2-weighted hyperintense signal (Figure 3).

**Figure 4:**
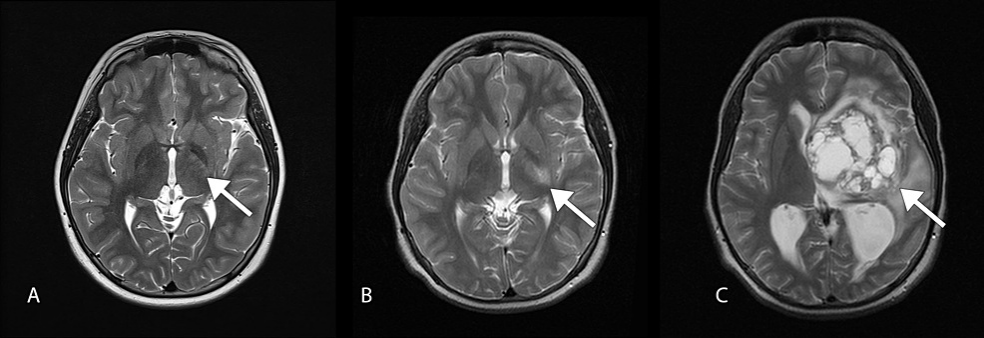
Axial T2-weighted image showing the temporal evolution of the left basal ganglia germinoma. Initial MRI in 2017 showing minimally hyperintense foci in the left basal ganglia (A, white arrow). In 2018, small cystic foci were noted in the same region of the left basal ganglia (B, white arrow). The latest MRI in 2020 showing progression into a large mass with multicystic appearance and midline shift to the right leading to obstructive hydrocephalus (C, white arrow). The mass is surrounded by perilesional white matter edema.

**Figure 5:**
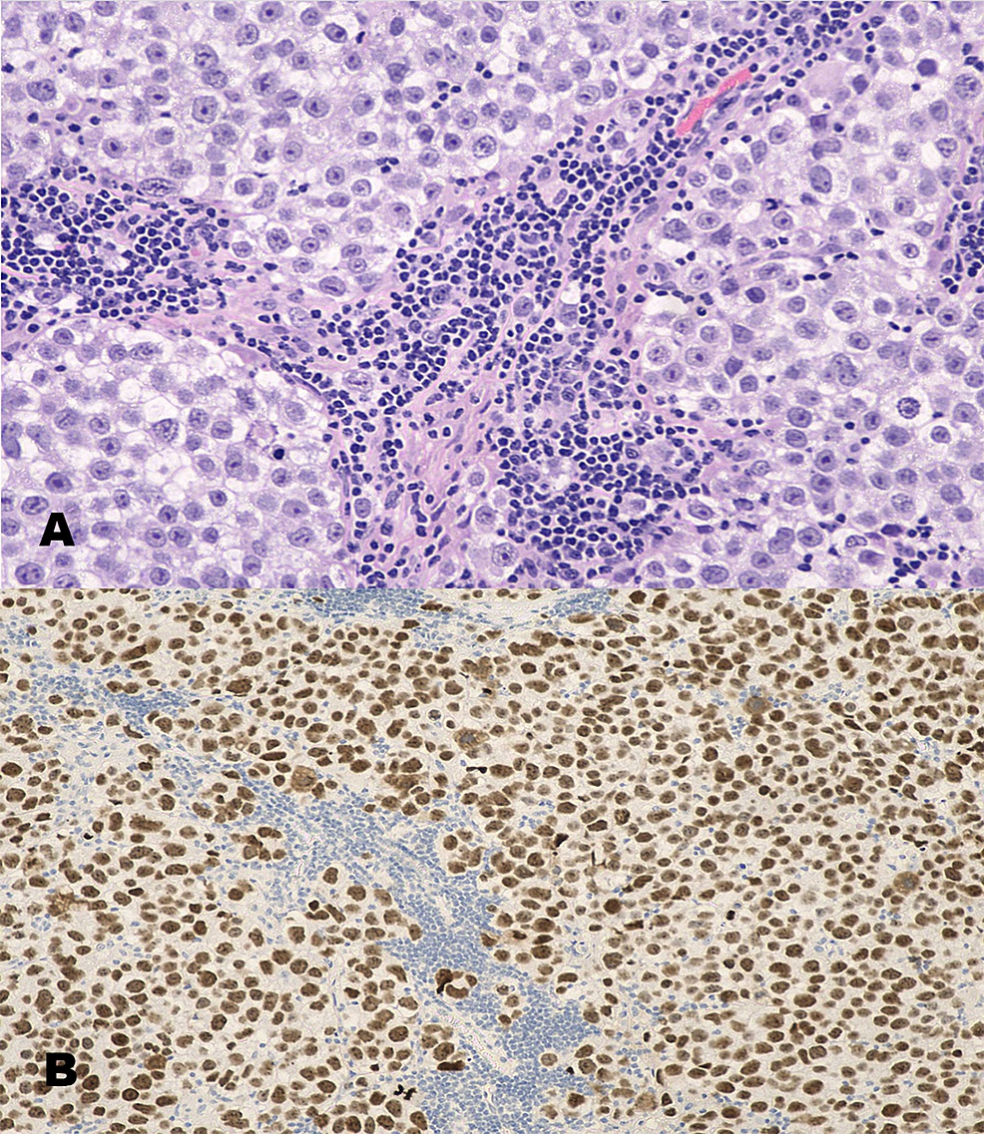
Histopathological specimens. (A) Hematoxylin and eosin stain (200×) shows tumor cells arranged in sheets and nests with intersecting fibrovascular septae of variable thickness. The fibrovascular septae are infiltrated by lymphocytes. (B) The germinoma cells show membranous staining with CD117 (100×).

Due to his right-sided hemiplegia and abnormal signal in the left basal ganglia, the patient was diagnosed as having an arterial ischemic stroke. However, his magnetic resonance angiography (MRA) showed no stenosis or abnormal signals in his intracranial vessels (Figure 3). Moreover, diffusion-weighted imaging (DWI) showed no evidence of restrictive diffusion. He was referred to a rehabilitation center and was able to perform his activities of daily living (ADL) independently. He remained stable for another two years. However, he again presented acutely with signs and symptoms of increased intracranial pressure, namely, vomiting, headache, and blurring of vision. Examination showed hypertonicity, hyperreflexia, and reduced power on the right side with grade 3/5, which was worse than before. He also had right homonymous hemianopia and diplopia. His pupils were unequal measuring 5 mm on the right and 4 mm on the left. His vital signs were stable. At this time, CT and MRI revealed a large solid-cystic mass in the left basal ganglia with mass effect and obstructive hydrocephalus (Figure 4). The patient underwent excision of the tumor, and histopathological examination revealed basal ganglia germinoma (Figure 5). There was no immediate postoperative complication and the patient was discharged. Currently, the patient is under close follow-up and has residual weakness. He also developed occasional seizures, which are under control with anti-epileptic drugs.

## Discussion

Germinomas mainly occur in intracranial midline structures such as the pineal and suprasellar regions. Rarely, they may arise from the basal ganglia. Intracranial GCTs are thought to arise during initial rostral neural tube development from the midline stream of totipotential cells. The development of the third ventricle may cause displacement of these cells from the midline, which can explain the manifestation of GCTs in ectopic locations [6]. The most common presenting symptom is hemiplegia. Other symptoms include headache, vomiting, mental retardation, and precocious puberty. However, these symptoms do not correlate with the average size of germinoma [7], as seen in our patient. His first presenting complaint was mild hemiparesis and did not correlate with the size of the tumor, which appeared as foci of hyperintense signal on T2-weighted imaging. Occasionally, serum alpha-fetoprotein and beta-subunit of human chorionic gonadotropin hormone may be detected in patients [8]. However, in our patient, the serum markers were only acquired after the diagnosis was made.

MRI shows early germinomas as ill-defined, homogenous, solid lesions without mass effect. In comparison to cortical gray matter, germinomas typically present as isointense to hyperintense on T2-weighted, T1-weighted, and fluid-attenuated inversion recovery images. Some also show a mild hypointense lesion concerning basal ganglia on T2-weighted imaging, likely due to iron deposition in the globus pallidus during adolescence. Contrast enhancement is not conspicuous in early imaging [3]. These MRI features were seen in our patient except for contrast enhancement which was not performed. These non-specific early imaging findings render the diagnosis of germinoma challenging. In non-contrast CT scans, early imaging findings include mild high-density lesions without mass effect. Additionally, contrast-enhanced CT may show an ill-defined tumor with cystic components which are frequently associated with calcification [9]. In our patient, CT showed mildly high density in the region of the left basal ganglia. However, there was no associated calcification, and contrast was not administered.

Okamoto et al. observed atrophy of the basal ganglia particularly in adolescents who presented with convulsions as an initial diagnostic sign of ectopic germinoma [10]. The postulated pathophysiology of basal ganglia atrophy includes direct tumor infiltration to the thalamus or internal capsule and autoimmune mechanisms comparable to Rasmussen’s encephalitis. It is also believed that the atrophy can be caused by Wallerian degeneration [10]. However, Wong et al. provided another postulation to the pathogenesis of hemiatrophy because the appearance of atrophy usually does not match with the size of the mass. They postulated gradual infiltration of the perforating arteries to the lenticulostriate arteries of the middle cerebral artery as the possible mechanism [11]. These features were not seen in our patient. There was no marked brain hemiatrophy, although the lateral ventricles were slightly asymmetrical. In addition, our MRA did not reveal any abnormal signal of the vessel nor there was any stenosis. Earlier studies using ^11^C-methionine positron emission tomography (MET-PET) showed high tracer uptake in ectopic germinomas which had no significant mass on conventional CT or MRI. MET-PET provides excellent contrast between the lesion and background normal brain tissue as the uptake is very low in the latter [12,13].

SWI is one of the latest methods to detect early basal ganglia glioma. SWI sequences can be used to detect blood products and biologic metal accumulation, as seen in intratumoral hemorrhage. The magnetic inhomogeneity causes the lesion to appear unusually larger on SWI than other conventional MRI sequences. In addition to hemorrhage, Lou et al. discussed the possibility of abnormal iron accumulation in basal ganglia germinoma as a factor causing the signal intensity seen on SWI [14]. This was evident in our patient who showed a blooming artifact on the first MRI in the region of the left basal ganglia.

Subsequent MRI revealed a slightly larger-sized, ill-defined, patchy hyperintense lesion in the left basal ganglia consistent with the study by Lee et al. who reported the development of intratumoral tiny cysts [3]. In contrast, our patient had no fluid within the cysts to suggest repeated intratumoral hemorrhage or necrosis due to tumor progression. Although the development of cysts is not specific to basal ganglia germinoma, it must be considered as a differential diagnosis, particularly in adolescent boys. The MRI protocol should be individualized for patients. Although the early subtle findings of ectopic germinomas were not seen, the repeat MRI should have administered contrast as there was a diagnostic dilemma. MRI showed the presence of enhancement in ill-defined patchy lesions [3]. When present, asymmetrical enhancement can raise the suspicion of lesions and narrow the differential diagnoses. The presence of small cysts in T2-weighted imaging can be seen in other conditions such as vasculitis, cavernous malformation, or, less likely, cerebral infarction related to this case.

Cerebral hemiatrophy is considered to be a result of Wallerian degeneration by direct tumor infiltration of the fiber tracts or into the ipsilateral perforating arteries [10,11]. Retrospectively, there was slight asymmetry in the patient’s lateral ventricle which progressed to merely a 3 mm difference, which may or may not be due to the presence of minimal atrophy. In advanced disease, basal ganglia germinoma has been described in the literature [7,9,15-17]. Typically, it can present as a heterogeneous mass and mostly contains hemorrhage, cyst, and necrosis. Our patient had a multicystic mass in the basal ganglia with few solid components. Hemorrhage is often depicted as a blooming artifact on gradient recall echo sequence. The solid component usually shows a relative isointense signal on T1-weighted images and iso or hypointense signal on T2-weighted images in comparison to gray matter, which is consistent with our patient. Variable contrast enhancement is usually observed, and restricted diffusion is one of the commonly seen features in basal ganglia germinoma, which were both demonstrated in the latest MRI of our patient. Despite the large size of the tumor, usually, there is minimal surrounding perilesional edema. The main differential diagnosis for basal ganglia tumors includes malignant glioma and lymphoma. However, these are difficult to differentiate on imaging alone. Thus, tissue biopsy is required for confirming the diagnosis.

## Conclusions

Basal ganglia germinoma is a rare tumor found in adolescents. It should be considered as a differential diagnosis when dealing with hemiplegia, especially in adolescent boys. Albeit challenging, early diagnosis is possible with awareness of the subtle changes seen on MRI such as a hyperintense signal on T2-weighted imaging, presence of hemiatrophy, and signal changes on SWI. Additionally, MET-CT may also be used for early diagnosis. The development of cysts on follow-up imaging can raise the suspicion of germinoma. When detected early, surgery may be avoided because this tumor is radio- and chemosensitive.
